# Data Mining Identifies Differentially Expressed Circular RNAs in Skeletal Muscle of Thermally Challenged Turkey Poults

**DOI:** 10.3389/fphys.2021.732208

**Published:** 2021-08-25

**Authors:** Kent M. Reed, Kristelle M. Mendoza, Juan E. Abrahante, Sandra G. Velleman, Gale M. Strasburg

**Affiliations:** ^1^Department of Veterinary and Biomedical Sciences, College of Veterinary Medicine, University of Minnesota, Saint Paul, MN, United States; ^2^University of Minnesota Informatics Institute, University of Minnesota, Minneapolis, MN, United States; ^3^Department of Animal Sciences, The Ohio State University, Ohio Agricultural Research and Development Center, Wooster, OH, United States; ^4^Department of Food Science and Human Nutrition, Michigan State University, East Lansing, MI, United States

**Keywords:** RNAseq, differential expression, circular RNA, turkey skeletal muscle, poult

## Abstract

Precise regulation of gene expression is critical for normal muscle growth and development. Changes in gene expression patterns caused by external stressors such as temperature can have dramatic effects including altered cellular structure and function. Understanding the cellular mechanisms that underlie muscle growth and development and how these are altered by external stressors are crucial in maintaining and improving meat quality. This study investigated circular RNAs (circRNAs) as an emerging aspect of gene regulation. We used data mining to identify circRNAs and characterize their expression profiles within RNAseq data collected from thermally challenged turkey poults of the RBC2 and F-lines. From sequences of 28 paired-end libraries, 8924 unique circRNAs were predicted of which 1629 were common to all treatment groups. Expression analysis identified significant differentially expressed circRNAs (DECs) in comparisons between thermal treatments (41 DECs) and between genetic lines (117 DECs). No intersection was observed between the DECs and differentially expressed gene transcripts indicating that the DECs are not simply the result of expression changes in the parental genes. Comparative analyses based on the chicken microRNA (miRNA) database suggest potential interactions between turkey circRNAs and miRNAs. Additional studies are needed to reveal the functional significance of the predicted circRNAs and their role in muscle development in response to thermal challenge. The DECs identified in this study provide an important framework for future investigation.

## Introduction

Efficient production of animal protein for human consumption is an important component of agriculture as animal protein = muscle = meat. Although various approaches have been investigated to increase production efficiency (Abo Ghanima et al., 2020; Alagawany et al., 2021), genetic selection for growth performance and the production of heavier market weight birds have increased the incidence of muscle diseases (myopathies) that cause significant losses to the industry (Mudalal et al., 2015; Velleman, 2015). For example, conditions such as White Striping and Woody Breast are becoming more common in the broiler industry (Petracci et al., 2013) and conditions such as pale, soft, exudative (PSE meat) are a concern for the turkey industry (Owens et al., 2000; Petracci et al., 2009; Zampiga et al., 2020). The genetic predisposition and etiology of these diseases are still poorly understood, although there is evidence of dysregulated lipid and glucose metabolism (Lake and Abasht, 2020).

Previous and ongoing studies are aimed at better understanding the effects that external factors, such as growth selection and temperature extremes have on gene expression and subsequent muscle development in poultry. Prolonged exposure to temperature extremes has detrimental effects on meat quality, including increased fat deposition, localized muscle fiber disorganization, and compromised protein functionality. For example, in studies of cultured turkey breast muscle satellite cells (SCs), exposure to thermal stress altered expression of adipogenic genes and increased lipid deposition (Clark et al., 2016; Clark and Velleman, 2016), suggesting potential conversion of satellite cells to an adipogenic lineage (Clark et al., 2017). *In vitro* study of proliferating and differentiating SCs from growth-selected lines compared to controls demonstrated a significant alteration in gene expression as a result of thermal challenge (Reed et al., 2017a, b). Expression of adipogenic genes by cultured SCs from commercial turkeys is more responsive to thermal challenge during proliferation than during differentiation (Xu et al., 2021a, b). Likewise, SCs from growth-selected turkeys are more sensitive to thermal stress compared to non-selected birds.

An *in vivo* study of newly hatched poults found the breast muscle of thermally challenged growth-selected birds responded through changes in gene expression predicted to have downstream transcriptional effects resulting in reduced muscle growth (Barnes et al., 2019). Non-selected birds responded through modulation of lipid-related genes, suggesting a reduction in lipid storage, transport and synthesis, consistent with changes in energy metabolism. In order to mitigate the incidence of myopathies and thereby improve meat quality and quantity, we need to better understand the cellular mechanisms that underlie muscle growth and development and how these are altered.

An emerging area in the study of gene regulation is circular RNAs (circRNAs). CircRNAs are novel, naturally occurring, single-stranded RNAs that are generated from exonic/intronic sequences joined head to tail and are widely expressed in eukaryotes (Wang et al., 2014; Patop et al., 2019). They lack polyA tails, function independently of ribosomes and rRNA, are resistant to RNA exonuclease (RNase R), and persist in the cell longer than mRNAs. Several mechanisms have been proposed for their generation (Qu et al., 2015), but they are identifiable in RNAseq data as back-spliced reads. Once thought to represent errors in RNA splicing, the abundance of circRNAs has been unappreciated in conventional bioinformatic analysis of genome-wide sequence data, but examination of this data with new algorithms has found circRNAs to be widely expressed (Liang et al., 2017; Xu et al., 2017). Although their functions are still poorly understood and mostly unknown, these RNAs appear to act as modifiers of gene expression by regulating transcription, RNA splicing, and translation by acting as microRNA (miRNA) sinks (Wilusz, 2018; Zhang et al., 2018; Kristensen et al., 2019). The closed loop structure of circRNAs makes them resistant to degradation by RNA exonuclease and thus more stable than linear RNAs. Their stability and abundance, especially in some body fluids, make them potential biomarkers (Zhang et al., 2018).

Based on sequence and expression analysis, circRNAs appear to be moderately conserved and expressed in a tissue and development-specific manner. Most are expressed at low levels, but some are expressed at levels higher than their linear RNA counterparts (Wilusz, 2018). Recent studies in several species, including some of agricultural importance, suggest that circRNAs modulate gene expression during myogenesis (Das et al., 2020) and play important roles in cell proliferation, differentiation, autophagy, and apoptosis (Kulcheski et al., 2016; Liang et al., 2017; Panda et al., 2017; Cao et al., 2018). For example, circRNAs are abundant and differentially expressed during chicken embryonic muscle development (Ouyang et al., 2018b), interact with innate response genes (Li et al., 2015; Zhao et al., 2015; Samir et al., 2016), and promote resistance to highly pathogenic J-strain avian leucosis virus (Zhang et al., 2017). Regarding muscle development, Ouyang et al. (2018a) identified a circRNA in the chicken supervillin gene (circSVIL) that could function as a sponge for miR-203, upregulating expression of c-JUN and MEF2C to promote the proliferation and differentiation of primordial muscle cells (myoblasts), a crucial process in muscle development. The objective of this study was to use a data-mining approach to characterize circRNA expression profiles within turkey skeletal muscle. This methodology was successfully used to characterize circRNAs in RNAseq data from humans and other model organisms (Memczak et al., 2013; Xu et al., 2017). Here, we used RNAseq data previously collected from a thermal challenge study of newly hatched turkey poults (Barnes et al., 2019). This first study of turkey skeletal muscle, identified 8924 unique circRNAs and significant differential expression was found in comparisons among thermal treatments and genetic lines.

## Materials and Methods

### circRNA Prediction and Expression Analysis

We used the original RNAseq sequence data of Barnes et al. (2019) for data mining (SRA BioProject PRJNA419215). In the challenge experiment, breast muscle tissues were harvested from hatchlings of two closed population lines (F and RBC2) exposed 3 days to reduced (31°C), elevated (39°C) or control temperature (35°C). The F-line was selected from the Randombred Control Line 2 (RBC2) only for 16-wk body weight and is faster growing (Nestor, 1977, 1984). The Randombred Control Line 2 (RBC2) represents a commercial bird from the late 1960s and has been maintained at The Ohio State University, Poultry Research Center (Wooster, OH) without conscious selection for any trait. Sequence data (RNAseq reads from 28 libraries, ∼18 million reads per library) was trimmed (Trimmomatic, Bolger et al., 2014) and quality-filtered (FastQC, Andrews, 2010). The resulting reads were mapped to the turkey genome (UMD5.1) using BWA-MEM (Li and Durbin, 2009). Circular RNAs were predicted using the CIRI software package (CIRI2, Gao et al., 2015), a multi-scan pipeline. The closest annotated gene to each predicted circRNA was obtained with BEDtools – closest option (Quinlan and Hall, 2010). Read counts for each circRNA were used for differential expression analysis using DESeq2 (CLC Genomics Workbench, v11.0.1).

### Confirmation of circRNAs

Confirmation of a set of predicted circRNAs was performed by PCR amplification and Sanger sequencing. For the selected circRNAs, flanking genome sequence was captured surrounding the splice site as indicated in CIRI. Oligonucleotide primer sets were designed for each circRNA using NIH-NCBI Primer-BLAST to target amplification of the circRNA junctions. The RNA samples were the same as originally used for RNAseq project (SRA BioProject PRJNA419215). Samples were first treated with RNase R that digests all linear RNA molecules except lariat or circular RNA structures. This depleted RNA was generated from 2 μg of total RNA using 5 units of RNase R exoribonuclease (Lucigen, Corp.) following manufacturer’s protocol (incubation reaction at 37°C for 20 min followed by 65°C for 20 min). Reverse transcription was performed with the Superscript IV kit (Invitrogen), 1 μg of RNase R depleted RNA and random hexamer priming as per manufacturer’s protocol (23°C for 10 min, 50°C for 10 min, followed by 80°C for 10 min). Aliquots of the resulting cDNA products were pooled as templates for PCR.

Amplification by PCR from the pool of RNase R depleted cDNA samples was conducted on 28 junction-flanking targets. PCR was performed using the Platinum Taq II system (Invitrogen) with 1 μl cDNA pool, and 0.4 μM each primer following manufacturer’s protocol. Thermal cycling conditions were as follows: 94°C for 2 min, then 30 cycles of 94°C for 15 s, 58°C for 15 s, 68°C for 30 s, followed by 10 min 72°C incubation. Products were resolved using 2% agarose electrophoresis and single products were selected for DNA sequencing. For sequencing, PCR products were purified using ExoSap-IT (Applied Biosciences) according to manufacturer’s protocol. Sanger sequencing was performed with both forward and reverse primers at the University of Minnesota Genomics Center core facility.

### Functional Prediction

Gene ontology (GO) overrepresentation tests were conducted with Panther v16.0 (Mi et al., 2021). Functional annotation clustering of the parental genes was performed with DAVID (Huang et al., 2009). Predicted circRNAs were scanned for miRNA binding/interaction sites through adapting application of miRDB (Liu and Wang, 2019).

## Results and Discussion

### circRNA Prediction

Sequence reads from the 28 paired-end libraries averaged 18.7 Million reads/library (Barnes et al., 2019). The software CIRI detects junction reads denoting circRNA candidates by differentiating and calculating the percentage of back-splice junction reads from non-junction reads. Detection of circRNAs is dependent on sequence depth with some commercial sequencing services recommending > 40M reads per sample. Analysis of the mapped reads and relative counts of back-splice junction and non-junction reads with CIRI2 predicted a total of 8924 unique circRNAs among the 28 libraries (Supplementary Table 1). On average, 3735.5 circRNAs were predicted per library and 2368.5 were shared between libraries within treatment groups (Table 1). Of the 8924 unique circRNAs, 1629 were shared across all 28 libraries.

**TABLE 1:** Summary of predicted circRNAs by treatment group^1^.

**Treatment Group**	**N**	**Avg # circRNAs**	**Common circRNAs**	**Average length (nt)**	**Average # junction reads**	**Average # non-junction reads**
31°C RBC2 [CS]	6	4362.8	2847	38670.8	56.2	786.3
31°C F-line [CF]	4	3365.3	2133	39170.8	40.2	259.1
35°C RBC2 [S cntl]	6	4155.7	2427	38835.3	54.0	623.2
35°C F-line [F cntl]	4	3496.5	2392	39232.0	38.3	294.7
39°C RBC2 [HS]	4	3266.8	2166	39080.8	36.6	321.5
39°C F-line [HF]	4	3326.0	2246	39028.0	37.3	326.4
Overall	28	3747.5	1629	38967.2	45.4	473.7

*^1^CS, cold-treated, slower growing (RBC2); CF, cold-treated, faster growing (F-line); HS, heat-treated, slower growing; and HF, heat-treated, faster growing.*

**FIGURE 1:**
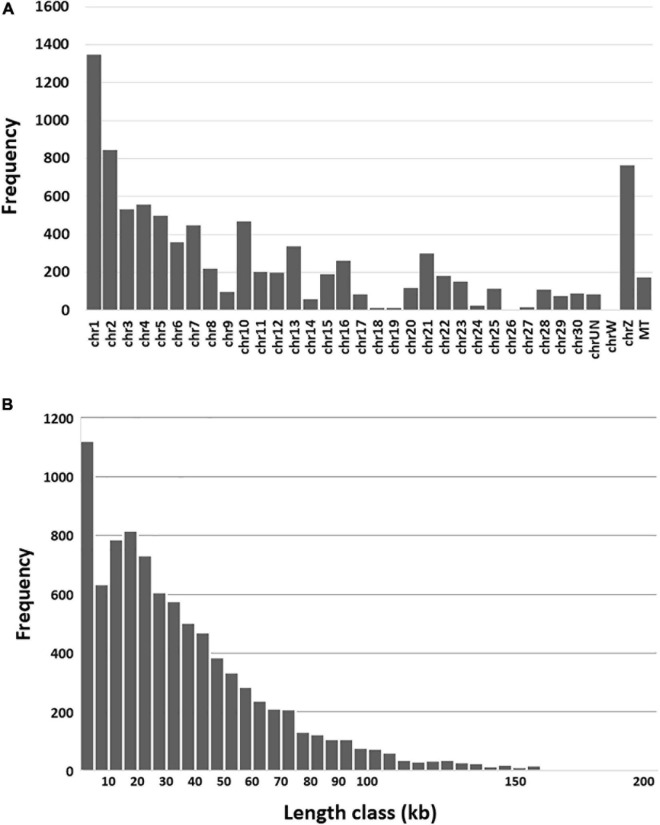
**(A)** Frequency of predicted circRNAs by chromosome. **(B)** Frequency of circRNAs by length class. Each vertical bar represents a window of 5 kb in length.

Genomic features including chromosome distribution, length and circRNA type were investigated. As expected the predicted circRNAs were distributed throughout the turkey genome and their frequency significantly corresponded to chromosome size (*p*-value < 0.00001, Figure 1A). Based on the position of the back-spliced reads in the genome, the predicted length of the circRNAs ranged from 134 bp to just under 200 kB (average 36.2 kB, Figure 1B). Using the current genome annotation (v102), the 8,924 predicted circRNAs were classified by CIRI2 as exonic (6.5%, average length 4,948 nt) or intronic (5.1%, average 23,543 nt). In addition, 88.4% fell outside of annotated genes and were designated as intergenic (average 39,289 nt). A common convention is to name circRNAs either in reference to their parental gene or identified function. Here we used the numbers assigned in the CIRI2 output to sequentially number the circRNAs along each chromosome progressing through the genome sequence (Supplementary Table 1).

The genome position is the defining circRNA feature since circRNA predictions are experiment-based and parental gene of origin may not be unique. For example, within the exonic class of circRNAs, 20 had 2 separate parental genes (Supplementary Table 1). Of these, 15 occurred where annotation of the two identified loci overlapped in the turkey genome. For the remaining 5 (circ0481, circ3782, circ4150, circ6709, and circ7308), the 2 genes were adjacent in the genome suggesting possible origin as chimeric RNA. Similarly, 20 “multigene” circRNAs were found in the intron class with 14 involving overlapping loci and 4 adjacent loci.

The 8924 circRNAs only mapped to a total of 4,513 parental or “closest” genes and a number of circRNAs had common parental genes. This is not uncommon in that some protein-coding genes generate multiple circRNAs through alternative circularization (Wilusz, 2018). Striking examples in our dataset include Myosin-7-like (*LOC104913726*) that had 35 associated circRNAs (circ6724-6758), and Thrombospondin 4 (*THBS4*) that had 33 (circ8115-8147). In the case of these multi-circRNA genes, individual circRNAs typically had different 3′ acceptor sites but common 5′ donors. Notable muscle genes with multiple circRNAs included the troponin genes (*TNNC2*, *TNNI2*, *TNNT2*, and *TNNT3*) which had 17, 14, 5, and 10 assigned circRNAs, respectively (Supplementary Table 1). The formation of circRNAs may be coupled to exon skipping events raising the potential that alternatively spliced genes may generate more circRNAs.

Functional annotation clustering of the parental genes found the cluster with the highest enrichment score (5.60) included genes in the GO categories Bioprocess (GO:0006412, Translation, Fold Enrichment = 2.52, FDR *p*-value = 1.40E-03), Molecular Function (GO:0003735, Structural constituent of ribosome, Fold Enrichment = 2.37, FDR *p*-value = 1.56E-03) and Cellular Component (GO:0022625, Cytosolic large ribosomal subunit, Fold Enrichment = 3.30, FDR *p*-value = 6.32E-03).

### Differential circRNA Expression

Expression of the predicted circRNAs was summarized for each of the six treatment groups of Barnes et al. (2019). These included the slower-growing RBC2 line (cold and heat treatments), the faster growing F-line (cold and heat treatments) and the F and RBC2 controls. Differentially expressed circRNAs (DECs) were classified by significance (FDR *p*-value) and observed fold change (| Log_2_FC| > 1.0 or > 2.0, Table 2).

**TABLE 2:** Numbers of differentially expressed circRNAs (DECs) in pairwise comparisons^1^.

	**Comparison**	**FDR *P* < 0.05**	**| Log_2_FC| > 1.0**	**| Log_2_FC| > 2.0**
**Temp**	CS (31R vs. 35R)	4	4	4
	HS (39R vs. 35R)	35	35	35
	CF (31F vs. 35F)	1	1	1
	HF (39F vs. 35F)	3	3	3
**Line**	31F vs. 31R	125	118	96
	35F vs. 35R	36	36	33
	39F vs. 39R	6	6	6

*^1^For each comparison of the treatment groups, the number of circRNAs with significant FDR *p*-value, and the numbers of significant circRNAs also with |Log_2_FC| > 1.0 and |Log_2_FC| > 2.0 are given.*

#### Temperature Effects

**FIGURE 2:**
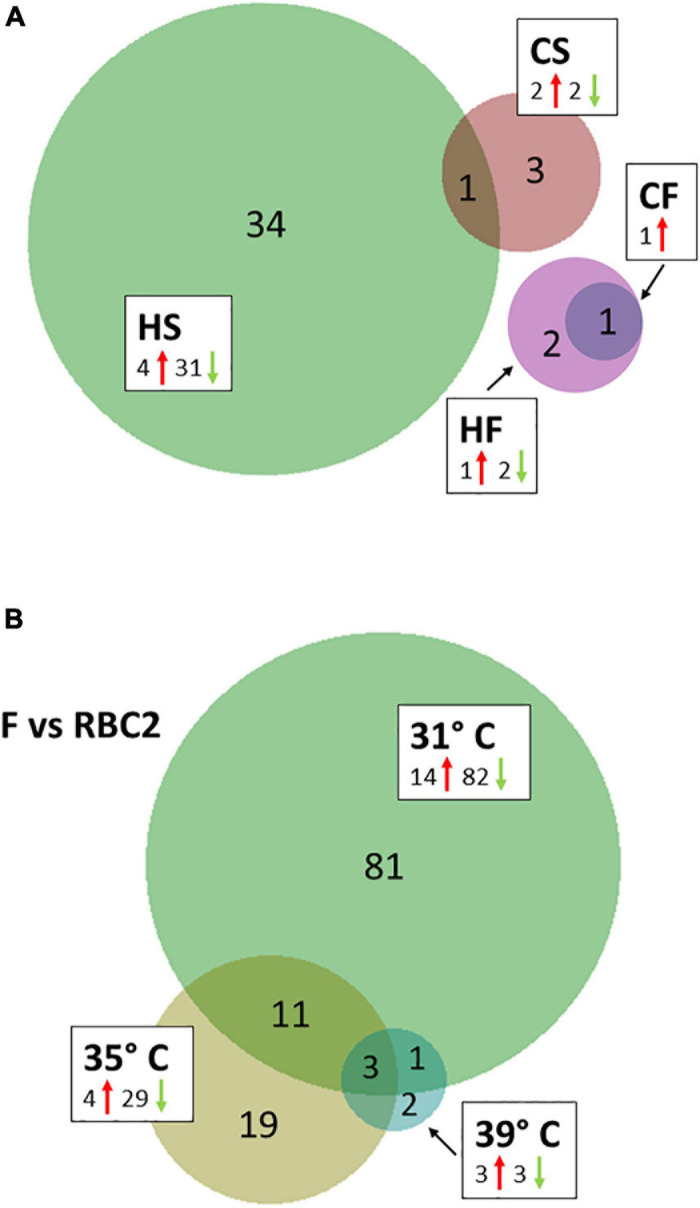
**(A)** Venn diagram of significant differentially expressed circRNAs (DEC) shared between temperature comparisons. HS, heat-treated slower growing (RBC2); CS, cold-treated slower growing; HF, heat-treated, faster growing (F-line); and CF, cold-treated, faster growing. **(B)** Distribution of significant differentially expressed circRNAs (DECs) in between-line comparisons.

Temperature effects on circRNAs were examined in 4 pairwise, within-line comparisons: CS = cold-brooded vs. control-brooded (31 vs. 35°C) slower growing poults (RBC2), HS = heat- vs. control-brooded (39 vs. 35°C) slower-growing poults (RBC2); CF = cold- vs. control-brooded (31 vs. 35°C) faster-growing poults (F-line); and HF = heat- vs. control-brooded (39 vs. 35°C) faster-growing poults (F-line). A total of 41 DECs that met the criteria of having FDR *p*-value < 0.05 and | Log_2_FC| > 2.0 was identified. The response of circRNAs in the breast muscle of turkey poults to thermal challenge was similar to the response in gene expression in that a greater number of circRNAs were differentially affected by heat than cold (Barnes et al., 2019). Four differentially expressed DECs were identified in the CS comparison (Tables 2, 3 and Figure 2A). Included were two significantly up regulated (circ3577 and circ7597) and two (circ6722 and circ3428) down regulated by the cold treatment. Three of the four were intergenic circRNAs with an average predicted length of 12,442 nt, and the fourth was exonic and 283 nt (Supplementary Table 1). Each of the associated genes are involved in muscle function and/or structure. Only a single DEC (circ5707, 1,445 nt) was identified in the CF comparison. This DEC was located in the intergenic region of tropomyosin 1 (alpha) and was up regulated by cold treatment. None of the DECs were in common between the two cold treatment comparisons (CS and CF). The greatest number of DECs was observed for the HS comparison (Table 3). These 35 DECs included 4 that were up regulated and 31 down regulated by the heat treatment. The majority (27) were classified as intergenic (average 27,961 nt) with 7 being intronic (average 18,852 nt) and 1 exonic (13,384 nt). One of the down regulated DECs (circ6722) was also similarly down regulated in the CS comparison. Lastly, 3 DECs were identified in the HF comparison, all of which were intergenic. The single up regulated DEC (circ5707) was also up regulated in the CF comparison.

**TABLE 3:** Significant differentially expressed circRNAs (FDR *P* < 0.05 and | Log_2_FC| > 2.0) in within-line treatment comparisons (see Figure 2A)^1^.

**CS (31R vs. 35R)**	**ID**	**Log_2_FC**	**FDR *p*-value correction**	**circRNA type**	**Gene ID**	**Description (Gene or closest gene)**
	circ3577	8.236988	0.000020	intergenic	PEX16	peroxisomal biogenesis factor 16
	circ7597	6.893736	0.001019	intergenic	LOC104914337	microtubule-actin cross-linking factor 1-like
	circ6722	−6.514929	0.000068	intergenic	LOC100543020	myosin-1B-like
	circ3428^2^	−8.688438	0.007392	exon	TNNT3	troponin T3, fast skeletal type
**CF (31F vs. 35F)**						
	circ5707	8.03131	0.00023	intergenic	TPM1	tropomyosin 1 (alpha)
**HS (39R vs. 35R)**						
	circ3950^2^	10.572252	0.000149	intergenic	LOC104911464	collagen alpha-2(I) chain-like
	circ8677	8.241432	0.001541	intergenic	N/A	
	circ8761	5.992206	0.008676	intergenic	ND1	NADH dehydrogenase subunit 1 (MT)
	circ1354	3.660428	0.013985	intergenic	LOC104909456	uncharacterized LOC104909456
	circ8066^2^	−5.585205	0.026434	intron	ARL15	ADP ribosylation factor like GTPase 15
	circ2219	−5.586529	0.031077	intergenic	LOC104910156	cadherin-12 pseudogene
	circ6362	−6.037131	0.015035	intergenic	LOC104913469	periplakin-like
	circ6722	−6.217346	0.017313	intergenic	LOC100543020	myosin-1B-like
	circ3571	−6.218754	0.007987	intergenic	LOC109367741	uncharacterized LOC109367741
	circ6927	−6.591872	0.021640	intergenic	LOC100550869	putative polypeptide N-acetylgalactosaminyltransferase-like protein 3
	circ4963	−6.740431	0.000273	intergenic	LOC109369148	uncharacterized LOC109369148
	circ0144	−6.746422	0.005320	intergenic	LOC104916543	voltage-dependent calcium channel subunit alpha-2/delta-1-like
	circ8470	−6.907068	0.000273	intergenic	LOC104915171	uncharacterized LOC104915171
	circ8000	−7.057952	0.001349	intergenic	LOC109363892	uncharacterized LOC109363892
	circ5692	−7.169252	0.000473	intergenic	TPM1	tropomyosin 1 (alpha)
	circ0134	−7.326608	0.001152	intergenic	LOC100541541	voltage-dependent calcium channel subunit alpha-2/delta-1-like
	circ6178	−7.376705	0.000579	intergenic	LOC109370008	ribosome biogenesis protein BOP1-like
	circ6222	−7.559996	0.000579	intergenic	TGFBI	transforming growth factor beta induced
	circ8482^2^	−7.649029	0.000026	exon	LOC100540511	leucyl-cystinyl aminopeptidase-like
	circ8493^2^	−7.758751	0.016605	intron	CPLX1	complexin 1
	circ4312	−7.824014	0.000336	intergenic	LOC104911721	E3 ubiquitin-protein ligase UBR3-like
	circ4482	−7.877863	0.000003	intergenic	ARL5A	ADP ribosylation factor like GTPase 5A
	circ8314^2^	−7.924424	0.000274	intron	LOC104915076	sarcoplasmic reticulum histidine-rich calcium-binding protein-like
	circ8230	−7.933418	0.000274	intergenic	LOC100547348	probable global transcription activator SNF2L2
	circ8106^2^	−8.289600	0.000004	intron	ADAMTS6	ADAM metallopeptidase with thrombospondin type 1 motif 6
	circ6755^2^	−8.356148	0.000418	intron	LOC104913726	myosin-7-like
	circ1325^2^	−8.706205	0.000130	intron	FCHSD2	FCH and double SH3 domains 2
	circ1326^2^	−8.752665	0.000000	intron	FCHSD2	FCH and double SH3 domains 2
	circ7382	−9.490423	0.000000	intergenic	GNB1	G protein subunit beta 1
	circ8071	−9.546974	0.000071	intergenic	PLPP1	phospholipid phosphatase 1
	circ8080	−9.620604	0.000068	intergenic	LOC100549854	superkiller viralicidic activity 2-like 2
	circ5155	−9.697864	0.000068	intergenic	MRPS14	mitochondrial ribosomal protein S14
	circ4471	−9.738572	0.000000	intergenic	LOC100546408	glycerol-3-phosphate dehydrogenase, mitochondrial
	circ5156	−10.292258	0.000000	intergenic	GPR52	G protein-coupled receptor 52
	circ6609	−10.748422	0.000034	intergenic	CRYM	crystallin mu
**HF (39F vs. 35F)**						
	circ5707	7.76196	0.03928	intergenic	TPM1	tropomyosin 1 (alpha)
	circ5262	−3.36306	0.03928	intergenic	LOC109369314	uncharacterized LOC109369314
	circ7568	−8.47206	0.03928	intergenic	TAF12	TATA-box binding protein associated factor 12

*^1^CS, cold-treated, slower growing (RBC2); CF, cold-treated, faster growing (F-line). HS, heat-treated, slower growing; and HF, heat-treated, faster growing.*

*^2^Denotes circRNA used in conformation studies.*

#### Line Effects

We also tested for interaction between brooding temperature and line effects by contrasting circRNA expression between the lines at each brooding temperature. For each comparison, the RBC2 line served as a control in contrast to the comparatively faster growing F-line. A total of 117 DECs was identified (FDR *p*-value < 0.05 and | Log_2_FC| > 2.0). At the control temperature (35°C) 33 DECs were observed between the two lines (Table 4 and Figure 2B). The majority (29) showed higher expression in the RBC2 group (down regulated in the F-line). Consistent with other comparisons, most of DECs were classified as intergenic with 5 intronic and 2 exonic. In the heat-treatment comparison, only 6 DECs were observed with 3 up regulated and 3 down regulated. The three down regulated DECs (circ3159, circ5016, and circ6976) were also significant and similarly differentially expressed in the control temperature comparison. These circRNAs and the up regulated circ3421 were also significant in the cold-treatment comparison. The greatest number of DECs was observed for the cold-treatment comparison where 96 circRNAs were differentially expressed. Again, the majority (82) were down regulated in the 16-wk bodyweight-selected F-line. Fourteen were shared with the control temp (35°C) and 4 with the heat-treated group (39°C). Differences in the circRNAs identified between the F and RBC2 lines can be attributed to past selection for 16 wk body weight and not genetic background differences as the F-line was derived from the RBC2 line (Nestor, 1977).

**TABLE 4:** Significant differentially expressed circRNAs (FDR *P* < 0.05 and | Log_2_FC| > 2.0) in within-temperature, between-line comparisons (see Figure 2B).

**35F vs. 35R**	**ID**	**Log_2_FC**	**FDR *p*-value correction**	**circRNA type**	**Gene ID**	**Description (Gene or closest gene)**
	circ0713	8.3015	5.067E-05	intergenic	PARVG	parvin gamma
	circ3297	5.7661	2.719E-03	intergenic	LOC104910961	uncharacterized LOC104910961
	circ3198	5.5322	4.901E-03	intergenic	LOC100542526	GDNF family receptor alpha-4
	circ1115	3.6854	2.491E-02	intergenic	LOC100539645	serine/threonine-protein kinase DCLK1
	circ6372	−2.6763	2.799E-02	intergenic	LOC104913349	rab11 family interacting protein 3-like
	circ2060	−3.2620	2.246E-03	intergenic	MLIP	muscular LMNA interacting protein
	circ1513	−5.2081	1.824E-02	intergenic	CEP170	centrosomal protein 170
	circ3945^1^	−5.7868	1.065E-02	exon	COL1A2	collagen type I alpha 2 chain
	circ4679	−5.9851	3.445E-02	intergenic	VTI1A	vesicle transport through interaction with t-SNAREs 1A
	circ6722	−6.2845	2.207E-03	intergenic	LOC100543020	myosin-1B-like
	circ4485	−6.6820	1.608E-04	intergenic	LOC109368621	uncharacterized LOC109368621
	circ4898	−6.6974	5.251E-03	intergenic	LOC109369187	uncharacterized LOC109369187
	circ6976	−6.7191	1.954E-04	intergenic	KIAA0753	KIAA0753 ortholog
	circ0856	−6.8527	1.382E-02	intergenic	LOC104917409	propionyl-CoA carboxylase alpha chain, mitochondrial-like
	circ3159	−7.0747	8.418E-03	intergenic	LOC100539657	protein Dok-7
	circ5784^1^	−7.3609	1.231E-11	intron	LOC104912917	SUMO-activating enzyme subunit 2-like
	circ6178	−7.4453	3.771E-04	intergenic	LOC109370008	ribosome biogenesis protein BOP1-like
	circ6222	−7.6286	3.771E-04	intergenic	TGFBI	transforming growth factor beta induced
	circ4397	−7.6612	2.413E-04	intergenic	LOC109368691	uncharacterized LOC109368691
	circ5016	−7.6705	1.539E-08	intergenic	LOC104909279	pancreatic alpha-amylase-like
	circ8493^1^	−7.8270	1.319E-02	intron	CPLX1	complexin 1
	circ4482	−7.9460	7.684E-07	intergenic	ARL5A	ADP ribosylation factor like GTPase 5A
	circ8618	−7.9526	4.300E-07	intergenic	HSD17B4	hydroxysteroid 17-beta dehydrogenase 4
	circ4490^1^	−8.0210	1.513E-02	exon	NEB	nebulin
	circ8106^1^	−8.3579	2.572E-07	intron	ADAMTS6	ADAM metallopeptidase with thrombospondin type 1 motif 6
	circ8383	−8.6867	2.572E-07	intergenic	LOC104915117	aminopeptidase O-like
	circ1325^1^	−8.7746	6.201E-05	intron	FCHSD2	FCH and double SH3 domains 2
	circ1326^1^	−8.8210	1.015E-07	intron	FCHSD2	FCH and double SH3 domains 2
	circ4470	−9.3925	5.067E-05	intergenic	LOC100546408	glycerol-3-phosphate dehydrogenase, mitochondrial
	circ7382	−9.5588	3.812E-09	intergenic	GNB1	G protein subunit beta 1
	circ8071	−9.6154	3.825E-05	intergenic	PLPP1	phospholipid phosphatase 1
	circ8080	−9.6890	3.421E-05	intergenic	LOC100549854	superkiller viralicidic activity 2-like 2
	circ6609	−10.8168	1.772E-05	intergenic	CRYM	crystallin mu
**31F vs. 31R**						
	circ3421	11.4585	5.867E-92	intergenic	LSP1	lymphocyte-specific protein 1
	circ1078	9.4738	1.276E-04	intergenic	LOC100546441	NEDD4-binding protein 2-like 2
	circ8521	7.5321	3.157E-03	intergenic	LOC104915210	maestro heat-like repeat-containing protein family member 7
	circ6921	7.1276	7.924E-03	intergenic	LOC109370644	uncharacterized LOC109370644
	circ7832^1^	6.9707	1.153E-02	exon	DOT1L	DOT1 like histone lysine methyltransferase
	circ7016	6.8980	1.297E-02	intergenic	LOC100545905	RNA-binding protein Musashi homolog 2-like
	circ0481^1^	6.5628	2.957E-02	exon	LOC109368814	uncharacterized LOC109368814
	circ4159^1^	6.3604	4.534E-02	exon	LOC109368509	uncharacterized LOC109368509
	circ7029	5.4093	4.555E-03	intergenic	LOC104913914	RNA-binding protein Musashi homolog 2-like
	circ5640	4.6731	8.392E-04	intergenic	LOC109369554	tight junction protein ZO-1-like
	circ7013	3.5407	3.836E-02	intergenic	LOC100545905	RNA-binding protein Musashi homolog 2-like
	circ7240	3.1543	1.116E-02	intergenic	LOC104914060	extracellular sulfatase Sulf-2-like
	circ4804	3.1081	3.932E-03	intergenic	PLS3	plastin 3
	circ1825	2.8058	4.344E-04	intergenic	LOC104909787	uncharacterized LOC104909787
	circ2223	−2.0787	2.734E-02	intergenic	LOC109366635	cadherin-12-like
	circ6890	−2.0827	1.197E-03	intergenic	LOC104913858	uncharacterized LOC104913858
	circ2439	−2.2317	1.606E-03	intergenic	LOC104910337	dystrobrevin alpha-like
	circ7142	−2.2967	3.716E-03	intergenic	LOC104913983	alpha-1-syntrophin
	circ0722	−2.5282	3.336E-02	intergenic	LOC104914802	motile sperm domain-containing protein 2-like
	circ5083	−2.5504	5.990E-03	intergenic	LOC104912332	myomegalin
	circ2697	−2.5580	4.482E-04	intergenic	LOC104910472	protein NDRG1-like
	circ3707	−2.6145	4.254E-02	intergenic	LOC100545111	homeobox protein Meis2
	circ4598	−2.7213	2.929E-02	intergenic	LOC104911901	uncharacterized LOC104911901
	circ2219	−2.8568	2.787E-02	intergenic	LOC104910156	cadherin-12 pseudogene
	circ2774	−2.8807	4.717E-02	intergenic	LOC104910674	electrogenic sodium bicarbonate cotransporter 1-like
	circ6222	−2.8891	1.043E-03	intergenic	TGFBI	transforming growth factor beta induced
	circ1295	−2.9772	9.808E-05	intergenic	ME3	malic enzyme 3
	circ5439	−3.0866	1.090E-02	intergenic	LOC104912562	uncharacterized LOC104912562
	circ1829	−3.2659	1.827E-02	intergenic	LOC109366437	uncharacterized LOC109366437
	circ0134	−3.4059	8.070E-05	intergenic	LOC100541541	voltage-dependent calcium channel subunit alpha-2/delta-1-like
	circ6719^1^	−3.4350	1.846E-02	intron	LOC100543020	myosin-1B-like
	circ4597	−3.5923	3.418E-03	intergenic	LOC104911901	uncharacterized LOC104911901
	circ8748	−3.6682	1.218E-08	intron	LOC104917234	uncharacterized LOC104917234
	circ6891	−3.6923	1.233E-03	intergenic	COL26A1	collagen type XXVI alpha 1 chain
	circ7880	−3.8744	3.451E-04	intergenic	LRG1	leucine rich alpha-2-glycoprotein 1
	circ8470	−4.2577	4.662E-07	intergenic	LOC104915171	uncharacterized LOC104915171
	circ4126	−4.9558	3.336E-02	intergenic	LOC100538839	homeobox protein Hox-A7
	circ2683^1^	−4.9973	4.901E-02	exon	COL22A1	collagen type XXII alpha 1 chain
	circ0078	−5.1580	2.787E-02	intergenic	LOC109371035	uncharacterized LOC109371035
	circ8549^1^	−5.1736	6.478E-03	intron	LOC104915227	transcription factor hamlet-like
	circ2821	−5.1798	2.617E-02	intergenic	LOC104910680	WD repeat and FYVE domain-containing protein 3-like
	circ7359	−5.1920	4.139E-02	intergenic	LOC104914128	sterile alpha motif domain-containing protein 11-like
	circ4283^1^	−5.2081	4.005E-02	intron	LOC100546506	sodium channel protein type 2 subunit alpha
	circ5189	−5.2322	1.297E-02	intergenic	LOC104912413	interleukin-23 receptor-like
	circ6371	−5.2433	4.504E-02	intergenic	RAB11FIP3	RAB11 family interacting protein 3
	circ3945^1^	−5.2905	1.449E-02	exon	COL1A2	collagen type I alpha 2 chain
	circ4384	−5.3516	3.932E-03	intergenic	LOC100550137	glycerol-3-phosphate dehydrogenase [NAD(+)], cytoplasmic-like
	circ8389	−5.4302	5.450E-03	intergenic	LOC104915117	aminopeptidase O-like
	circ6679	−5.4377	3.418E-03	intergenic	SIRT4	sirtuin 4
	circ7975	−5.5721	3.279E-03	intergenic	LOC104914837	ran-binding protein 3-like
	circ4757	−5.6362	1.271E-02	intergenic	LOC104912085	uncharacterized LOC104912085
	circ2684	−5.6568	1.449E-02	intergenic	COL22A1	collagen type XXII alpha 1 chain
	circ2385	−5.6730	1.980E-03	intergenic	LOC109366741	uncharacterized LOC109366741
	circ1832	−5.6818	3.892E-02	intergenic	LOC104909788	eyes absent homolog 4-like
	circ4194^1^	−5.8571	3.654E-03	exon	RPL37A	ribosomal protein L37a
	circ3680	−5.8805	2.377E-04	intergenic	LOC104911146	uncharacterized LOC104911146
	circ0081	−5.9175	3.654E-03	intergenic	LOC109371035	uncharacterized LOC109371035
	circ5118	−5.9869	2.025E-03	intergenic	PBX1	PBX homeobox 1
	circ3080	−6.0371	2.549E-03	intergenic	LOC104910777	uncharacterized LOC104910777
	circ7597	−6.7250	5.450E-03	intergenic	LOC104914337	microtubule-actin cross-linking factor 1-like
	circ8543	−6.7620	1.878E-07	intron	SLC44A1	solute carrier family 44 member 1
	circ8284	−6.7651	2.010E-08	intergenic	LOC104915053	focadhesin-like
	circ0144	−6.7776	1.218E-08	intergenic	LOC104916543	voltage-dependent calcium channel subunit alpha-2/delta-1-like
	circ5259	−6.8364	3.564E-05	intergenic	LOC109369314	uncharacterized LOC109369314
	circ4679	−6.8541	8.655E-09	intergenic	VTI1A	vesicle transport through interaction with t-SNAREs 1A
	circ6472	−6.8723	1.194E-06	intergenic	LOC109370229	uncharacterized LOC109370229
	circ6976	−6.8977	2.519E-05	intergenic	KIAA0753	KIAA0753 ortholog
	circ5050	−6.9198	4.228E-10	intergenic	LOC100546512	serine/threonine-protein kinase Nek7-like
	circ7908	−6.9447	1.287E-09	intergenic	LOC104914743	dymeclin-like
	circ7008	−6.9532	4.370E-02	intergenic	LOC109370664	uncharacterized LOC109370664
	circ5684	−7.1293	5.600E-05	intergenic	TPM1	tropomyosin 1 (alpha)
	circ3159	−7.2314	1.975E-03	intergenic	LOC100539657	protein Dok-7
	circ8482^1^	−7.4103	7.336E-06	exon	LOC100540511	leucyl-cystinyl aminopeptidase-like
	circ3121	−7.7263	4.994E-17	intergenic	RAB28	RAB28, member RAS oncogene family
	circ6919	−7.8457	1.552E-06	intergenic	SSTR2	somatostatin receptor 2
	circ1405	−7.9434	2.260E-17	intergenic	LOC100542006	heterogeneous nuclear ribonucleoprotein L-like
	circ8229	−7.9890	4.344E-04	intergenic	LOC100547348	probable global transcription activator SNF2L2
	circ8623	−8.0447	4.055E-04	intergenic	LOC104915350	methylcrotonoyl-CoA carboxylase beta chain, mitochondrial-like
	circ3577	−8.0673	4.344E-04	intergenic	PEX16	peroxisomal biogenesis factor 16
	circ3430^1^	−8.1215	1.271E-02	exon	TNNT3	troponin T3, fast skeletal type
	circ4482	−8.1278	9.600E-21	intergenic	ARL5A	ADP ribosylation factor like GTPase 5A
	circ5016	−8.2775	3.826E-21	intergenic	LOC104909279	pancreatic alpha-amylase-like
	circ8314^1^	−8.3226	2.501E-20	intron	LOC104915076	sarcoplasmic reticulum histidine-rich calcium-binding protein-like
	circ0133	−8.7052	3.152E-08	intergenic	LOC104917538	voltage-dependent calcium channel subunit alpha-2/delta-1-like
	circ0974^1^	−8.7561	1.808E-04	intron	LOC104917520	TSC22 domain family protein 3 pseudogene
	circ8230	−8.7562	1.532E-23	intergenic	LOC100547348	probable global transcription activator SNF2L2
	circ8493^1^	−8.8250	5.544E-08	intron	CPLX1	complexin 1
	circ8106^1^	−8.8852	2.213E-20	intron	ADAMTS6	ADAM metallopeptidase with thrombospondin type 1 motif 6
	circ4490^1^	−8.8859	2.005E-19	exon	NEB	nebulin
	circ1324^1^	−9.0991	1.939E-08	intron	FCHSD2	FCH and double SH3 domains 2
	circ8383	−9.4757	5.645E-21	intergenic	LOC104915117	aminopeptidase O-like
	circ1325^1^	−9.5920	3.688E-35	intron	FCHSD2	FCH and double SH3 domains 2
	circ6752^1^	−10.3964	9.463E-05	intron	LOC104913726	myosin-7-like
	circ8080	−10.3975	2.678E-51	intergenic	LOC100549854	superkiller viralicidic activity 2-like 2
	circ5156	−10.9400	2.678E-51	intergenic	GPR52	G protein-coupled receptor 52
	circ6609	−11.4190	2.852E-82	intergenic	CRYM	crystallin mu
**39F vs. 39R**						
	circ3421	10.6966	4.1548E-49	intergenic	LSP1	lymphocyte-specific protein 1
	circ4471	9.3665	1.8383E-31	intergenic	LOC100546408	glycerol-3-phosphate dehydrogenase, mitochondrial
	circ3041	6.5192	1.1626E-05	intergenic	LOC109367350	uncharacterized LOC109367350
	circ6976	−6.3908	1.5806E-02	intergenic	KIAA0753	KIAA0753 ortholog
	circ5016	−6.9386	1.8050E-10	intergenic	LOC104909279	pancreatic alpha-amylase-like
	circ3159	−6.9528	1.2651E-03	intergenic	LOC100539657	protein Dok-7

*^1^Denotes circRNA used in conformation studies.*

Given the larger number of DECs identified in the between-line comparisons of turkey poults we performed overrepresentation tests (Panther v16.0, Mi et al., 2021) to test for functional clustering of the parental genes. In the control temperature (35°C) group comparison, analysis of the 33 DECs found significant overrepresentation for GO categories of extracellular matrix (GO:0031012) and extracellular matrix organization (GO:0030198) (Table 5). Similarly, analysis of the 96 DECs in the cold temperature (31°C) comparison, also found significant overrepresentation for extracellular matrix categories (GO:0030198 and GO:0031012) but also actin cytoskeleton and cellular component organization and the cellular component (supramolecular fiber, GO:0099512). This finding contrasts results from the transcriptome where the slower growing RBC2 birds responded to thermal stress primarily with changes in lipid-related genes (Barnes et al., 2019). Muscle of F-line hatchlings responded to thermal stress through changes in genes involved in ubiquitination and modulators of gene expression, with a predicted reduction in muscle growth. Interaction networks among the transcribed RNAs (mRNA, miRNA, circRNA and other non-coding RNAs) and the response of these networks to physiologic stressors merit further investigation.

**TABLE 5:** Summary of PANTHER Overrepresentation Test of the parental genes of differentially expressed circRNAs (DECs) in between-line comparisons of turkey poults after heat exposure^1^.

	**Category**	***Gallus gallus* – REFLIST genes (17887)**	**Observed turkey genes**	**Expected**	**Fold Enrichment**	***P*-value**
**35F vs. 35R**	BP-extracellular matrix organization (GO:0030198)	131	4	0.11	36.41	6.98E-03
	CC-extracellular matrix (GO:0031012)	262	4	0.22	18.21	2.48E-02
**31F vs. 31R**	BP-extracellular matrix organization (GO:0030198)	131	5	0.17	29.68	1.25E-03
	BP-actin cytoskeleton organization (GO:0030036)	271	5	0.35	14.35	3.96E-02
	BP-cellular component organization (GO:0016043)	2454	16	3.16	5.07	2.70E-06
	CC-extracellular matrix (GO:0031012)	262	5	0.34	14.84	8.29E-03
	CC-supramolecular fiber (GO:0099512)	352	6	0.45	13.26	2.01E-03

*^1^Turkey gene IDs were matched to the chicken gene reference list for analysis in PANTHER Mi et al., 2021. For each comparison the Gene Ontology category [Biological Process (BP) or Cellular Component (CC)], the number of genes in the reference list and those differentially expressed in the turkey are given. Fold enrichment is the number of DEC parental genes divided by number Expected and only those greater than 2.0 are given. All *p*-values are as determined by the binomial statistic.*

Finally, we compared the DECs from both temperature and line comparisons to the significant differentially expressed genes (DEGs) identified for the same treatment comparisons in the original RNAseq study (Barnes et al., 2019). No intersection was observed between the DEC gene IDs and the DEGs (| Log_2_FC| > 2.0). We also compared the directionality of expression differences for DECs in the exonic and intronic classes (clearly defined parental genes) with the parental gene transcript expression. In most cases, expression of the parental gene transcripts were essentially invariant (| Log_2_FC| < 0.5) and in many of the treatment comparisons, the directionality of change was opposite that observed for the DECs. Only a single parental gene (LOC100540511, leucyl-cystinyl aminopeptidase-like) in the 31F vs. 31R comparison showed significant expression changes in both the circRNA (circ8482, Log_2_FC = −7.41, FDR *p*-value = 7.34E-06) and parental gene transcript (Log_2_FC = −1.25, FDR *p*-value = 0.037). This indicates that the differences in circRNA expression are not just a function of expression changes in the parental genes.

### Confirmation of circRNAs

Although we identified thousands of putative circRNAs, confirmation of these predictions with orthogonal techniques is necessary prior to further functional interpretation. As circRNAs are computationally predicted on an experiment-level basis, performing this for all predicted circRNAs would be daunting. In this respect, differential expression analysis can help focus and guide these efforts toward the set of circRNAs that are impacted by the treatments of interest. We selected 28 circRNAs for confirmation by a PCR-based approach designed to specifically target the splice junction. The circRNAs for analysis were selected to included representatives from the exonic and intronic classes (Supplementary Table 2), varied ranges in predicted length (143 to 49,826 nt), with the majority being differentially expressed in the experimental comparisons. Primers were designed to produce fragments of approximately 250 bp with an average of 125 bp of 5′ and 3′ flanking sequence. Using pooled cDNA synthesized from RNase R depleted RNA, 23 of 28 predicted circRNA junctions were amplified by PCR. Of the 23 amplified products, 14 cleanly sequenced through and confirmed the predicted back-spliced junction. The remaining 9 either produced very faint PCR products or multiple bands that could not be directly sequenced (Supplementary Table 2). This reiterates the necessity for orthogonal confirmation. The 5 circRNA junctions that did not amplify could represent linear RNAs present in the original RNA samples but not resistant to RNase R (false positives). Ideally, characterization of circRNAs should include independent sequencing of libraries created from depleted RNA (RNase R digested) to help eliminate false positives and identify minor classes of circRNAs with low expression. This work is ongoing in our laboratory.

### Functional Analysis of circRNAs

The function of individual circRNAs appears to be diverse (Zhang et al., 2018). Some have been shown to function as miRNA sponges or through binding endogenous competing RNAs, whereas others interact with RNA binding proteins and mRNAs to regulate alternative splicing and/or transcription (Huang et al., 2020). Some circRNAs remain in the nucleus interacting with Pol II machinery to regulate expression of their parental genes while others may have a role in protein translation including the potential for coding proteins (Abe et al., 2015; Bose and Ain, 2018; Lei et al., 2020). Unlike differentially expressed gene transcripts (DEGs) identified in traditional RNAseq studies, the functional significance of DECs (circRNAs) are more difficult to discern in that it is dependent on potential interactions with other RNA molecules and not necessarily tied to function of the parental gene of origin.

We used computational methods to assess the potential for the turkey circRNAs to interact with one class of small RNA molecules, miRNAs. As “sponges” circRNAs sequester miRNAs reducing the number of freely available interacting molecules and thereby suppressing their activity on target genes. Likewise, binding of RNA-binding proteins could also regulate gene expression. Scanning circRNAs for miRNA target sites was instrumental in identifying the novel RNA interactions involving the mouse *Sry* (Memczak et al., 2013) and human *CDR1* genes (Hansen et al., 2011). The now classic example of RNA sponge activity is the circular antisense CDR1 transcript in humans that contains > 70 binding site seed matches for miR-17 (Hansen et al., 2011). Unfortunately, a miRNA database is currently not available for the turkey and therefore we relied on comparative analyses. As such, detailed analysis of the potential significance of circRNA differential expression and downstream effects on RNA interactions (such as miRNA binding) are limited.

**FIGURE 3:**
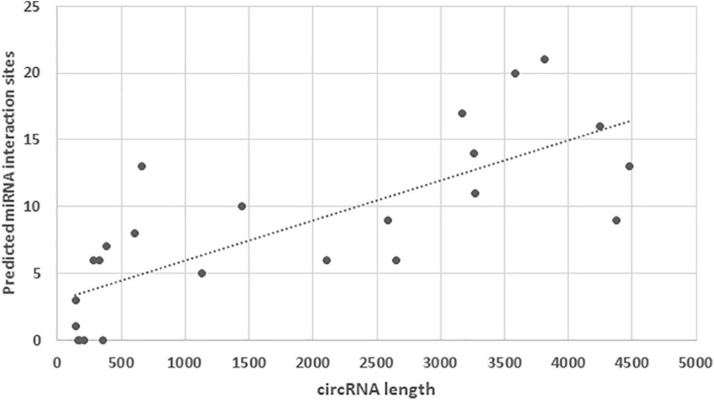
Distribution of miRNA target sites identified within the 24 DECs with lengths predicted by miRDB of less than 5000 nt.

To explore potential interactions, we used miRDB (Liu and Wang, 2019) and the miRNA dataset of the closely related chicken to scan for miRNA binding sites within the turkey circRNAs. Because extremely long RNAs are penalized within the miRDB algorithm (Xiaowei Wang, pers com), we selected the 24 DECs with predicted lengths of less than 5000 nt for analysis. Target sites for miRNAs were identified in 20 of the DECs with an average of 8.4 sites per circRNA (Figure 3). As might be expected the number of target sites increased positively with circRNA length. The greatest number of target sites (21) was identified in circ1354 (3811 nt) with miRNA gga-miR-6677-3p having the highest target score (80). Within the miRDB database, this chicken miRNA has 713 predicted gene targets. The highest miRNA target score (91) was observed for interaction of circ6609 with gga-miR-7437-3p. A total of 9 predicted miRNA target sites was identified for this 2487 nt circRNA, which was significantly underrepresented in the HS poult comparison. Understanding the potential interactions is complicated as within miRDB database a total of 740 predicted gene targets are included for gga-miR-7437-3p.

We also examined two of the circRNAs of the multi-circRNA troponin T3 (TNNT3) gene. The exonic circRNA (circ3428) derived from TNNT3 is one of the smaller predicted circRNAs at 283 nt and it was significantly down regulated (Log_2_FC = −8.69) in the CS treatment comparison (Table 3). A second exonic DEC (circ3430, 327 nt) with sequence overlap with circ3428 within TNNT3, was also significantly downregulated in the F-Line under cold treatment (Log_2_FC = −8.12). These short circRNAs had 13 and 12 predicted miRNA target sites, respectively, with highest target scores corresponding to miRNAs gga-miR-7483-3p (83) and gga-miR-7437-5p (74). These miRNAs have 204 and 714 predicted target genes in the chicken, respectively. Interestingly, the junction regions of both of these circRNAs could be amplified by PCR, but failed in sequencing. We attribute the difficulty in sequencing to the likelihood that multiple amplicons were generated from these overlapping circRNAs. There is increasing evidence for the importance of miRNA interactions in muscle biology. For example, miR-24 (3p) and miR-128 (3p) play a role in myogenic satellite cell migration in turkey with a potential impact on muscle growth and development (Harding and Velleman, 2016; Velleman and Harding, 2017). Interactions between miRNAs and circRNAs have the potential to alter these processes (Liang et al., 2017; Ouyang et al., 2018a) and the circRNAs identified herein provide a framework for generating new hypotheses.

## Conclusion

As circRNAs have not been previously characterized for any tissue in the turkey, we have only just begun to explore potential implications. Here we demonstrated the ability to identify circRNAs that are abundant and differentially expressed in turkey skeletal muscle in response to thermal stress. Analysis of a subset of these confirmed their presence and resistance to RNase R digestion and indicates presence of functional sequence elements within the predicted circRNAs. Characterization of the predicted circRNAs is complicated by their abundance and also potentially by completeness of the turkey genome. The majority of the circRNAs identified in this study, as defined by the junction reads, encompassed genomic regions > 10 Kb that included sequence gaps. Therefore, we suspect that this average length is potentially biased and necessitates confirmation of gaps by PCR and sequencing. Identifying differentially expressed circRNAs provides a framework for future investigation and detailed molecular and physiologic studies are needed to reveal their functional significance.

## Data Availability Statement

Publicly available datasets were analyzed in this study. This data can be found here: SRA BioProject PRJNA419215.

## Ethics Statement

Data used in this study was previously collected from experiments performed under institutionally approved protocol.

## Author Contributions

All authors have made a substantial and intellectual contribution to the work, and approved the submitted article. KR conceived and designed the experiments with GS and SV. KM and JA performed the experiments and statistical analysis. KR wrote the manuscript. SV, GS, KM, and JA revised the manuscript.

## Conflict of Interest

The authors declare that the research was conducted in the absence of any commercial or financial relationships that could be construed as a potential conflict of interest.

## Publisher’s Note

All claims expressed in this article are solely those of the authors and do not necessarily represent those of their affiliated organizations, or those of the publisher, the editors and the reviewers. Any product that may be evaluated in this article, or claim that may be made by its manufacturer, is not guaranteed or endorsed by the publisher.
